# Unraveling trajectories from aplastic anemia to hematologic malignancies: genetic and molecular insights

**DOI:** 10.3389/fonc.2024.1365614

**Published:** 2024-03-13

**Authors:** Namsoo Kim, Yu Jeong Choi, Seung-Tae Lee, Jong Rak Choi, Chuhl Joo Lyu, Saeam Shin, June-Won Cheong

**Affiliations:** ^1^ Department of Laboratory Medicine, Yonsei University College of Medicine, Seoul, Republic of Korea; ^2^ Dxome Co. Ltd., Seongnam-si, Gyeonggi-do, Republic of Korea; ^3^ Department of Pediatric Hematology-Oncology, Yonsei Cancer Center, Severance Hospital, Yonsei University College of Medicine, Seoul, Republic of Korea; ^4^ Division of Hematology, Department of Internal Medicine, Yonsei University College of Medicine, Severance Hospital, Seoul, Republic of Korea

**Keywords:** aplastic anemia, secondary cancer, hematologic malignancy, genomic profile, clonal evolution

## Abstract

**Background:**

Aplastic anemia (AA), characterized by hematopoietic stem cell deficiency, can evolve into different hematologic malignancies. Our understanding of the genetic basis and mechanisms of this progression remains limited.

**Methods:**

We retrospectively studied 9 acquired AA patients who later developed hematologic malignancies. Data encompassed clinical, laboratory, karyotype, and next-generation sequencing (NGS) information. We explored chromosomal alterations and mutation profiles to uncover genetic changes underlying the transition.

**Results:**

Nine AA patients developed myelodysplastic syndrome (seven patients), acute myeloid leukemia (one patient), or chronic myelomonocytic leukemia (one patient). Among eight patients with karyotype results at secondary malignancy diagnosis, monosomy 7 was detected in three. Trisomy 1, der(1;7), del(6q), trisomy 8, and del(12p) were detected in one patient each. Among three patients with NGS results at secondary malignancy diagnosis, *KMT2C* mutation was detected in two patients. Acquisition of a *PTPN11* mutation was observed in one patient who underwent follow-up NGS testing during progression from chronic myelomonocytic leukemia to acute myeloid leukemia.

**Conclusion:**

This study highlights the genetic dynamics in the progression from AA to hematologic malignancy. Monosomy 7’s prevalence and the occurrence of *PTPN11* mutations suggest predictive and prognostic significance. Clonal evolution underscores the complexity of disease progression.

## 1 Introduction

Aplastic anemia (AA) is a rare but potentially life-threatening disorder characterized by a profound deficiency of hematopoietic stem cells (HSCs) in the bone marrow, leading to insufficient production of red blood cells, white blood cells, and platelets (1). This condition results in pancytopenia, a state characterized by reduced levels of all major blood cell types in the peripheral blood (2). AA’s etiology encompasses both acquired and inherited factors (3). Acquired AA, which accounts for the majority of cases, often develops as a result of immune-mediated destruction of HSCs (4). On the other hand, inherited forms of AA, such as Fanconi anemia, are caused by genetic mutations that disrupt DNA repair pathways and lead to bone marrow failure (5).

The management of AA has evolved significantly over the years (6). Historically, the prognosis was grim, with limited therapeutic options (7). However, advances in medical science have resulted in innovative treatment strategies. Hematopoietic stem cell transplantation from a matched sibling donor has been considered the gold standard for treatment, offering a potential cure (8, 9). However, the availability of suitable donors is limited, and the procedure carries risks of complications (3). As an alternative, immunosuppressive therapy has emerged as a viable option (10). Employing agents such as antithymocyte globulin and cyclosporine, immunosuppressive therapy aims to mitigate the immune response leading to hematopoietic cell destruction (11). This approach has shown promise, particularly in non-severe cases, leading to hematologic improvement and prolonged survival (12). While clonal evolution to conditions such as myelodysplastic syndrome (MDS), acute myeloid leukemia (AML), and other hematologic malignancies have been subject of investigation, our understanding of the genetic mechanism of progression from AA to other hematologic malignancies remains incomplete (13).

While clonal evolution, secondary cancers, and the progression to leukemia have garnered some research attention in the context of AA (14), there is a gap in the understanding of the long-term trajectory toward different hematologic malignancies. Moreover, few comprehensive studies have examined the chromosomal alterations and results from next-generation sequencing (NGS) in these cases (15–17). To address these gaps, this study sought to analyze the less-explored aspects of AA evolution into secondary hematologic malignancy. We sought to investigate the clonal evolution mechanism in AA by presenting the genetic profile of patients diagnosed with secondary hematologic malignancies.

## 2 Methods

### 2.1 Patients and data

This retrospective study enrolled acquired AA patients who underwent bone marrow examination at the time of diagnosis of secondary hematologic malignancy between January 2013 and August 2023 within a single institution. Inherited AA was excluded from analysis. Their clinical data and laboratory test results, including those of genetic analysis, were collected and analyzed. Demographic and laboratory data at the time of AA diagnosis and information about treatments received for AA were included. Based on their complete blood count and bone marrow results at diagnosis, patients were classified into non-severe AA, severe AA, and very severe AA (17). Additionally, we enrolled eight patients diagnosed with AA who underwent long-term follow-up, including bone marrow aspiration, biopsy, chromosome karyotyping, and NGS.

We collected data on the time interval from initial AA diagnosis, complete blood count, bone marrow findings, karyotype, and NGS results at the time of secondary hematologic malignancy diagnosis. Treatment for secondary malignancy was also documented. Outcomes and follow-up were assessed, with outcomes categorized as progression, alive, dead, or follow-up loss. Additional follow-up duration was documented for patients classified as alive or dead. This study protocol was approved by the Institutional Review Board of Yonsei University Health System (4-2023-0926).

### 2.2 Cytogenetic and molecular genetic analyses

The G-banding karyotyping procedure was conducted on heparinized bone marrow aspirate according to standard protocols. At least 20 metaphases were evaluated, and the karyotype was described according to the International System for Human Cytogenetic Nomenclature. For targeted NGS, a custom set of probes (Dxome Co. Ltd., Gyeonggi-do, Korea) focusing on 531 genes associated with hematologic neoplasms was employed (**Supplementary Table S1**). Genomic DNA extracted from the diagnostic bone marrow aspirate was used to create libraries, which were then subjected to hybridization with capture probes and sequenced using NextSeq 550Dx from Illumina (San Diego, CA, USA). All steps were performed as per the manufacturer’s guidelines. NGS data analysis was conducted using a DxSeq analyzer (Dxome). Single-nucleotide variants, small insertions and deletions, and copy number variants were identified according to previously described methods (18, 19). Confirmation of germline variant was achieved with NGS results using skin fibroblast analysis. Identified variants were categorized into four tiers based on the Association for Molecular Pathology’s guidelines, which involve contributions from the American College of Medical Genetics and Genomics, the American Society of Clinical Oncology, and the College of American Pathologists (20). The classification process involved consultation of web databases such as OncoKB and cBioPortal (21, 22). The accuracy of all variants was confirmed visually using the Integrated Genomics Viewer.

## 3 Results

### 3.1 Patient demographics

Nine patients were enrolled in this study (**Table 1**). Among the nine patients for whom laboratory data were available at the time of AA diagnosis, three had severe AA and five had non-severe AA. Six of the 9 enrolled patients underwent AA treatment, with danazol treatment being the most common. Patients were diagnosed with secondary hematologic malignancy on average 13.6 years (range, 3.5–34 years) after AA diagnosis. Seven patients were diagnosed with MDS, and one patient each was diagnosed with AML and chronic myelomonocytic leukemia (CMML). During treatment of secondary hematologic malignancy, two patients underwent allogeneic hematopoietic stem cell transplantation, and five patients were treated with decitabine, azacitidine, or danazol. Two patients (one MDS patient and one CMML patient) later progressed to AML.

**Table 1:** Patient characteristics.

Patient	Initial diagnosis	Age at AA diagnosis	Sex	Laboratory data at AA diagnosis			Treatment for AA	Secondary malignancy	Period of latency	Laboratory data at secondary malignancy diagnosis			Treatment for secondary malignancy	Outcome/follow-up
				ANC (×10^9^/L)	ARC (×10^9^/L)	Platelets (×10^9^/L)				ANC (×10^9^/L)	ARC (×10^9^/L)	Platelets (×10^9^/L)		
P1	NSAA	31Y	F	4200	NA	75	Danazol	MDS-RS-MLD	22y2m	220	17.1	27	Decitabine	Dead/4m
P2	SAA	50Y	F	700	19.8	10	Steroid, IST	MDS-MLD	4y5m	3140	296	48	Decitabine	Progressed to AML
P3	NSAA	21Y	M	1130	52.2	262	None	MDS-MLD	4y1m	780	25.2	324	Allo-HSCT	Alive/1y1m
P4	NSAA	54Y	F	1310	63.4	90	Steroid, danazol	AML	11y2m	60	21.2	19	Allo-HSCT	Dead/1m
P5	SAA	62Y	F	760	28.4	<3	Eltrombopag	CMML-2	3y6m	1680	178	1038	Decitabine	Progressed to AML
P6	SAA	60Y	F	360	12	30	Steroid, danazol	MDS-EB1	21y5m	1360	58.3	40	Azacitidine	Alive/1m
P7	NSAA	76Y	M	640	NA	53	None	MDS-MLD	5y2m	1100	92.7	63	Follow-up Loss	Follow-up loss
P8	NSAA	39Y	F	4710	71.1	31	Danazol	MDS-MLD	16y6m	6870	107.8	191	Danazol	Alive/3y8m
P9	AA	4Y	F	NA	NA	NA	None	MDS-MLD	34y	400	30.6	113	Follow-up	Alive/2y2m

AA, aplastic anemia; allo-HSCT, allogenic hematopoietic stem cell transplantation; AML, acute myeloid leukemia; ANC, absolute neutrophil count; ARC, absolute reticulocyte count; CMML-2, chronic myelomonocytic leukemia-2; IST, immunosuppressive therapy; M, male; m, month; MDS-MLD, myelodysplastic syndrome with multilineage dysplasia; MDS-RS-MLD, myelodysplastic syndrome with ring sideroblasts with multilineage dysplasia; NA, not available; NSAA, non-severe aplastic anemia; SAA, severe aplastic anemia; y, year.

Additionally, we enrolled eight patients diagnosed with AA who underwent long-term follow-up, including bone marrow aspiration, biopsy, chromosome karyotyping, and NGS, and analyzed their results (**Supplementary Table S3**). Except for one, all showed a normal karyotype. Among the eight patients, six did not have tier 1/2 mutations in bone marrow DNA NGS. One patient had a tier 2 somaticmutation in the *WRAP53* gene, while another had multiple somatic mutations in *TP53* and *TET2* with low variant allele frequency (VAF) of less than 1%.

### 3.2 Genetic analysis at diagnosis of secondary hematologic malignancy

**Figure 1:**
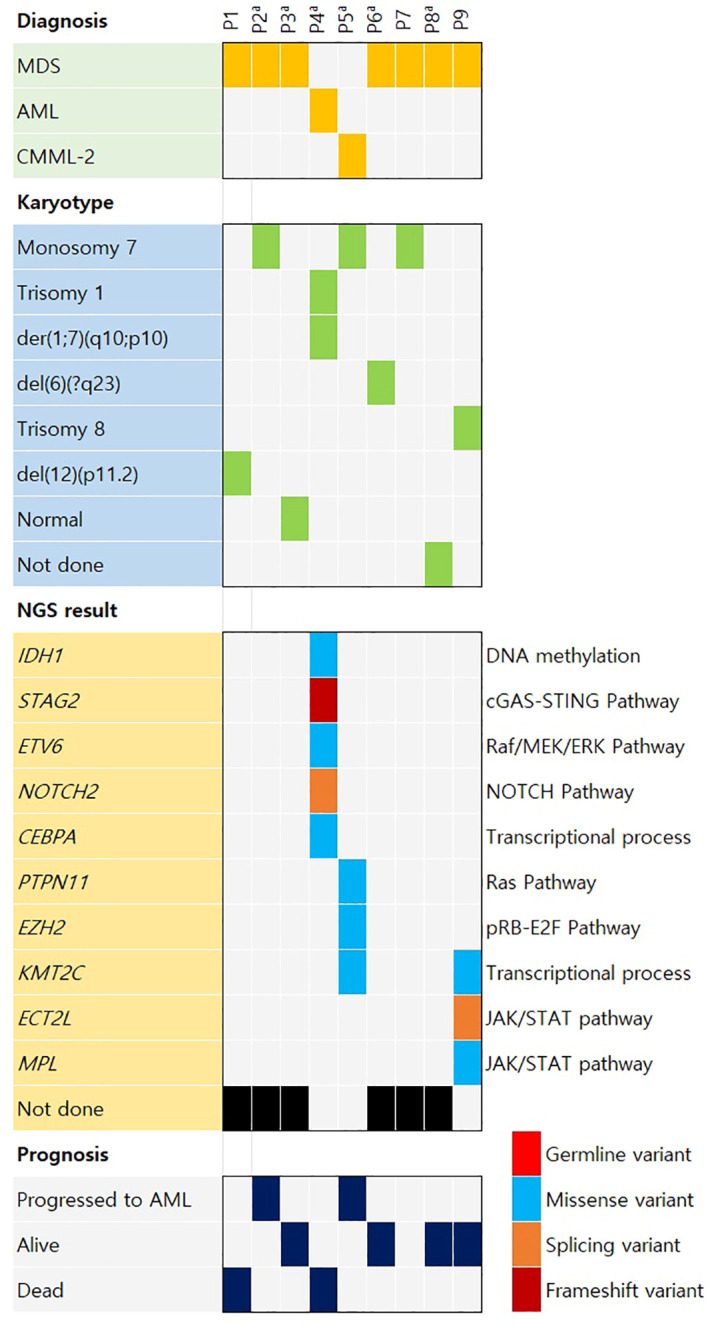
Oncoplot of the case distribution of aplastic anemia patients who later developed hematologic malignancy. AML, acute myeloid leukemia; CMML, chronic myelomonocytic leukemia; MDS, myelodysplastic syndrome. ^a^Six patients (P2–P6 and P8) showed normal karyotype at aplastic anemia diagnosis. The other three patients (P1, P7, and P9) had no information on karyotype at aplastic anemia diagnosis.

An oncoplot was created for the type of hematologic malignancy, karyotype results, NGS results, and prognosis of the patients (**Figure 1**). Six patients who underwent karyotype analysis at the time of AA diagnosis (P2–P6, P8) showed normal karyotype. Among the nine patients who underwent karyotype testing at diagnosis of secondary hematologic malignancy, monosomy 7, the most frequent abnormality, was detected in three patients, and no duplicate variants were found for the other abnormalities. NGS results collected at diagnosis of secondary malignancy were obtained from four patients. *KMT2C* mutations were detected in two patients, and *IDH1*, *STAG2*, *ETV6*, *NOTCH2*, *CEBPA*, *PTPN11*, *EZH2*, *KMT2C*, *ECT2L*, and *MPL* mutations were detected in one patient each. In three patients, an average of three somatic mutations (range, 0–5 mutations) was identified. NGS results are provided in **Supplementary Table S2**.

### 3.3 Clonal evolution in patients who progressed to AML

**Figure 2:**
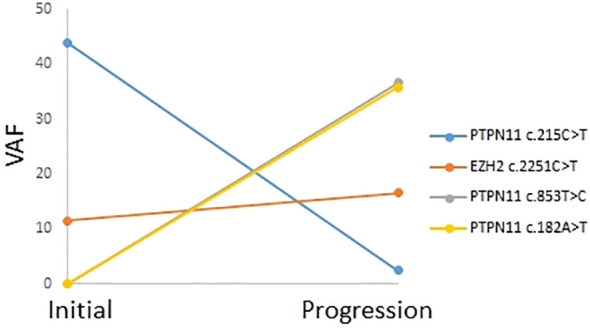
Mutation profile and variant allele frequencies for tier 1 and tier 2 variants in patient P5, assessed through two next-generation sequencing tests for chronic myelomonocytic leukemia-2 and progression to acute myeloid leukemia.

Patients P5 underwent two NGS tests, when diagnosed with secondary hematologic malignancies (CMML-2 for P5) and when they progressed to AML. The changes in VAF for tier 1 and 2 variants that were confirmed to be somatic are shown in **Figure 2**. *PTPN11* c.215C>T and *EZH2* c.2251C>T variants were present at CMML-2 diagnosis. One of the *KMT2C* mutations in P5 (c.12140G>T) exhibited a high VAF in both NGS tests, and it was unclear whether it overlapped with monosomy 7 as a germline mutation or if it was a somatic mutation. Upon progression to AML, the VAF of the *PTPN11* c.215C>T variant decreased, and two new *PTPN11* variants (c.852T>C and c.182A>T) with high VAFs appeared, while the VAF of the *EZH2* c.215C>T variant slightly increased.

## 4 Discussion

The presented study provides valuable insights into the less-explored aspects of the evolution of AA into various hematologic malignancies. The journey of AA patients toward different malignancies is a complex process influenced by genetic, molecular, and environmental factors (23). The findings from this study contribute to our understanding of the underlying mechanisms and molecular alterations associated with this progression.

The study’s focus on cytogenetic analysis and NGS provides a window into the genetic changes that occur during the progression of AA to hematologic malignancy. The identification of chromosomal abnormalities and somatic variants, such as the monosomy 7 abnormality detected in a significant proportion of patients, highlights the importance of these alterations in disease evolution. The emergence of mutations during disease progression, as demonstrated by P2 and P5, suggests clonal evolution and implies that certain genetic events might drive the transformation to AML.

Understanding the molecular changes associated with disease progression has potential clinical implications. The emergence of gene variants during progression could serve as potential biomarkers for predicting the risk of transformation to aggressive forms of hematologic malignancy (14). Early identification of these variants might enable tailored monitoring and intervention strategies to mitigate the risk of disease evolution.

We compared nine patients diagnosed with AA who were subsequently diagnosed with a different hematologic malignancy to eight patients who were not diagnosed with such malignancies. Among the nine patients, chromosome abnormalities were observed in seven, whereas only one of the eight patients exhibited chromosome abnormalities. Furthermore, among the three patients diagnosed with hematologic malignancies who underwent NGS, tier 1/2 mutations with high VAF were observed in two, whereas only one of the eight patients exhibited tier 1/2 mutations with high VAF. These observations suggest that progression to hematologic malignancy involves factors such as chromosome abnormalities and gene mutations. Thus, it is evident that NGS should be included in the follow-up tests for patients diagnosed with AA.

In this study, not all patients underwent chromosomal karyotyping at diagnosis, with only 6 out of 9 patients being tested, all of whom showed a normal karyotype. This aligns with existing literature suggesting that only 5-15% of patients diagnosed with AA exhibit an abnormal karyotype (24, 25). However, when considering the 9 patients who experienced relapse, all except one who did not undergo testing and one who showed a normal karyotype exhibited abnormal karyotypes. This presents a significant difference and underscores the necessity of chromosomal karyotyping for patients diagnosed with secondary hematologic malignancies originating from AA.

Monosomy 7 has been recognized as a significant genetic anomaly in MDS and AML (26). Monosomy 7 is known to occur in approximately 5%–20% of cases in MDS and AML (27), and, in our patient cohort, it was observed in an even greater proportion of patients than usual (44%). Furthermore, monosomy 7 is associated with adverse prognosis in MDS (28); among the three patients in this study with trackable outcomes of the four patients diagnosed in total, all progressed to AML, corroborating this association. Previous studies have also linked a higher prevalence of monosomy 7 to the karyotypes of high-risk severe AA patients (17), implying that aggressive treatment and management might be warranted for AA patients harboring monosomy 7.

In the existing literature, somatic mutations of AA were reported to occur in genes such as *PIGA*, *BCOR/BCORL1*, *DNMT3A*, and *ASXL1 *(29). However, our analysis of data from progressed patients revealed slightly different results. Remarkably, among the three patients who progressed from MDS/CMML to AML, both patients who underwent NGS testing exhibited *PTPN11* mutations. *PTPN11* mutations are present in approximately 7%–12% of AML cases, and those in AML patients with wild-type NPM1 are associated with adverse patient outcomes (30). Interestingly, in our study, the *PTPN11* mutations might have played a driver role in patient diagnosis of hematologic mutations. However, there is scarce research on the relationship between AA and the *PTPN11* gene. It is essential to explore the presence of driver mutations and genetically linked prognostic factors through NGS at the time of diagnosis not only in AA patient cohorts, but also in the broader context of hematologic diseases. NGS has greatly advanced our understanding and management of aplastic anemia by allowing comprehensive genetic analysis, facilitating the discovery of novel mutations, and enabling personalized treatment strategies based on individual genetic profiles.

Additionally, we identified mutations in the *IDH1* and *EZH2* genes in our patients. *IDH1*, along with *TP53*, has been reported as a gene associated with an increased risk of AML. It is noteworthy that our patient initially diagnosed with AA were later diagnosed with AML with high VAF of *IDH1*, aligning with the disease’s trajectory (31). *EZH2* is implicated in various hematologic malignancies, with literature suggesting its relevance in proposing novel therapeutic directions for AA (32). Indeed, among observed cases, we have witnessed patients transitioning from AA to CMML and then to AML, emphasizing the necessity of incorporating genes associated with hematologic malignancies into our panel.

While the study provides valuable insights, certain limitations should be acknowledged. The retrospective nature of this study and its small patient cohort might limit the generalizability of the findings. Since the majority of patients have not received immunosuppressive therapy, recent studies on disease progression may deviate slightly from the current trajectory. There were no patients who underwent NGS at the time of diagnosis with AA. Further, the absence of comprehensive karyotype results for all patients and the focus on specific genes in the NGS analysis might miss other relevant genetic alterations.

## 5 Conclusion

In conclusion, this study delves into the complex journey of AA patients as they progress toward various hematologic malignancies. The genetic and molecular insights gained from cytogenetic analysis and NGS offer a glimpse into the underlying mechanisms of disease evolution. The findings have the potential to guide future research, improve risk stratification, and contribute to the development of targeted therapies for these patients. As we continue to unravel the intricate pathways of disease progression, collaborative research endeavors will be crucial in advancing our understanding and improving the outcomes of patients with AA and its associated complications.

## Data availability statement

The datasets presented in this study can be found in online repositories. The names of the repository/repositories and accession number(s) can be found in the article/**Supplementary Material**.

## Ethics statement

This study protocol was approved by the Institutional Review Board of Yonsei University Health System (4-2023-0926). The studies were conducted in accordance with the local legislation and institutional requirements. The ethics committee/institutional review board waived the requirement of written informed consent for participation from the participants or the participants’ legal guardians/next of kin because only test results of participants were used.

## Author contributions

NK: Writing – original draft, Data curation, Conceptualization. YJC: Writing – review & editing. S-TL: Writing – review & editing, Methodology. JRC: Writing – review & editing, Resources, Investigation. CJL: Writing – review & editing, Resources, Investigation, Conceptualization. SS: Writing – review & editing, Supervision, Data curation, Conceptualization. J-WC: Writing – review & editing, Supervision, Resources, Investigation.
