# Supplementary material for: Unraveling trajectories from aplastic anemia to hematologic malignancies: genetic and molecular insights
Source: Front Oncol. 2024 Mar 13;14:1365614. doi: 10.3389/fonc.2024.1365614 (PMC10965666; doi:10.3389/fonc.2024.1365614)
Supplement: Supplementary file 1 [file Table_1.docx]

Supplementary Material

**Supplementary Table S1.** List of the 531 genes utilized in the study's gene panel.

*ABCB7, ABCG5, ABCG8, ABL1, ABL2, ACD, ACTB, ACTN1, ACVRL1, ADA, ADAMTS13, AIRE, AK1, AK2, AKT2, ALAS2, ALDOA, AMN, ANK1, ANKRD26, ANO6, AP3B1, ARID1A, ARPC1B, ASXL1, ATG2B, ATM, ATR, ATRX, AXIN1, BCL11B, BCL2, BCL6, BCOR, BCORL1, BIRC3, BLM, BLOC1S3, BLOC1S6, BPGM, BRAF, BRCA1, BRCA2, BRCC3, BRIP1, BTG1, BTK, BTLA, C15orf41, C1QA, C1QB, C1QC, C1R, C1S, C2, C3, C4A, C4B, C4BPA, C5, C6, C7, C8A, C8B, C9, CALR, CARD11, CASP10, CBL, CBLB, CCND1, CD200, CD247, CD27, CD36, CD3D, CD3E, CD40LG, CD46, CD58, CD59, CD79B, CDAN1, CDKN1B, CDKN2A, CDKN2B, CEBPA, CFB, CFD, CFH, CFHR1, CFHR2, CFHR3, CFHR4, CFHR5, CFI, CFP, CHD9, CLPB, CNOT3, COL3A1, COL4A1, COL4A2, COX4I2, CREBBP, CRLF2, CSF1R, CSF3R, CST3, CTC1, CTCF, CUBN, CUX1, CXCR4, CYB5R3, CYBA, CYBB, CYCS, DCLRE1C, DDX41, DGKE, DHFR, DIAPH1, DIS3, DKC1, DNAJC21, DNM2, DNMT1, DNMT3A, DPAGT1, DTNBP1, EBF1, ECT2L, EED, EFL1, EGFR, EGLN1, EGLN2, EIF2AK4, ELANE, ENG, EP300, EPAS1, EPB41, EPB42, EPCAM, EPO, EPOR, ERCC4, ERCC6L2, ERG, ETNK1, ETV6, EZH2, F10, F11, F12, F13A1, F13B, F2, F2R, F5, F7, F8, F9, FAM46C, FANCA, FANCB, FANCC, FANCD2, FANCE, FANCF, FANCG, FANCI, FANCL, FANCM, FAS, FASLG, FAT1, FBXW7, FCGR3B, FERMT3, FGA, FGB, FGG, FLI1, FLT3, FOXP3, FYB, G6PC3, G6PD, GATA1, GATA2, GATA3, GBA, GCLC, GFI1, GFI1B, GGCX, GIF, GINS1, GLA, GLRX5, GNAS, GNB1, GP1BA, GP1BB, GP6, GP9, GPI, GPRC5A, GPX1, GSR, GSS, HAX1, HBA1, HBA2, HBB, HBD, HFE, HIF1A, HK1, HOOK1, HOXA11, HPS1, HPS3, HPS4, HPS5, HPS6, HRAS, HRG, HSPA9, ID3, IDH1, IDH2, IFNG, IFNGR1, IKZF1, IKZF2, IKZF3, IL2RB, IL2RG, IL7R, IRF1, ITGA2, ITGA2B, ITGB2, ITGB3, ITK, JAGN1, JAK1, JAK2, JAK3, KCNK3, KDM5C, KDM6A, KIF23, KIT, KLF1, KLKB1, KMT2A, KMT2C, KMT2D, KNG1, KRAS, LAMB4, LAMTOR2, LCK, LIG4, LMAN1, LMNA, LMO1, LPIN2, LRP1B, LUC7L2, LYL1, LYST, MAD2L2, MAGT1, MAP2K1, MAP2K2, MASTL, MBL2, MCFD2, MECOM, MEF2B, MEFV, MET, MKL1, MLH1, MLPH, MMACHC, MMADHC, MPL, MRE11, MSH2, MSH6, MTAP, MTHFR, MTR, MTRR, MUT, MVK, MYB, MYC, MYD88, MYH9, MYO5A, MYSM1, NAF1, NBEAL2, NBN, NCF2, NCOR2, NF1, NFE2, NHEJ1, NHP2, NLRP3, NOP10, NOTCH1, NOTCH2, NPM1, NR3C1, NRAS, NSD2, NT5C2, NT5C3A, NTRK3, NUP214, P2RX1, P2RY12, PALB2, PARN, PAX5, PBX1, PDGFRA, PDGFRB, PDHA1, PDHX, PFKL, PFKM, PGK1, PGM3, PHF6, PICALM, PIEZO1, PIGA, PIK3CD, PIK3R1, PKLR, PLA2G4A, PLA2G7, PLAT, PLAU, PLG, PMS2, PNP, POT1, PPM1D, PRF1, PRKACG, PROC, PROCR, PROS1, PRPF40B, PTEN, PTK2B, PTPN11, PTPN2, PTPRC, PTPRD, PUS1, RAB27A, RAC1, RAC2, RAD21, RAD50, RAD51, RAD51C, RAF1, RAG1, RAG2, RASGRP2, RB1, RBBP6, RBM8A, RELN, RFWD3, RHAG, RHOA, RIT1, RPL10, RPL11, RPL15, RPL26, RPL27, RPL31, RPL35A, RPL36, RPL5, RPS10, RPS14, RPS15, RPS17, RPS19, RPS24, RPS26, RPS27, RPS27A, RPS28, RPS29, RPS7, RTEL1, RUNX1, RUNX1T1, SAMD9, SAMD9L, SBDS, SEC23B, SERPINC1, SERPIND1, SERPINE1, SERPINF2, SERPING1, SETBP1, SETD2, SF1, SF3A1, SF3B1, SH2B3, SH2D1A, SHOC2, SLC11A2, SLC19A2, SLC25A38, SLC2A1, SLC35C1, SLC37A4, SLC4A1, SLCO1B1, SLCO1B3, SLFN14, SLX4, SMAD4, SMAD9, SMARCD2, SMC1A, SMC3, SMPD1, SOS1, SPINK5, SPRED1, SPTA1, SPTB, SRC, SRP54, SRP72, SRSF2, STAG1, STAG2, STAT3, STAT5B, STEAP3, STIM1, STN1, STX11, STXBP2, SUZ12, SYNE1, TAL1, TAZ, TBL1XR1, TBX1, TBXA2R, TBXAS1, TCF3, TCIRG1, TEC, TERC, TERF2IP, TERT, TET1, TET2, TET3, TFPI, THBD, THPO, TINF2, TLX1, TLX3, TMPRSS6, TNFAIP3, TNFRSF13B, TNFRSF14, TNFRSF1A, TOX, TP53, TPI1, TRAF3, TREX1, TRNT1, TSLP, TSR2, TUBB1, TYK2, U2AF1, U2AF2, UBE2T, UGT1A1, UNC13D, USB1, VHL, VIPAS39, VKORC1, VPS13B, VPS33B, VPS45, VWF, WAS, WDR1, WIPF1, WRAP53, WT1, XBP1, XIAP, XK, XRCC2, YARS2, ZAP70, ZRSR2*

**Supplementary Table S2.** Variants identified at diagnosis of secondary hematologic malignancies.

| Patient ID | NGS# | Diagnosis | Gene | Transcript number | NT alteration | AA alteration | Type | VAF (%), Depth | Tier |
| --- | --- | --- | --- | --- | --- | --- | --- | --- | --- |
| P4 | 1 | AML | *IDH1* | NM_001282387.1 | c.394C>T | p.Arg132Cys | Missense | 15.4,  3007 | Tier 1 |
|  |  |  | *STAG2* | NM_001042749.1 | c.3324_3327del | p.Gln1108HisfsTer7 | Frameshift | 12.4,  2054 | Tier 2 |
|  |  |  | *ETV6* | NM_001987.4 | c.1256T>G | p.Phe419Cys | Missense | 30.3,  1714 | Tier 3 |
|  |  |  | *NOTCH2* | NM_024408.3 | c.-5A>G | - | Splicing | 9.7,  3685 | Tier 3 |
|  |  |  | *CEBPA* | NM_004364.3 | c.568T>C | p.Ser190Pro | Missense | 6.9,  2042 | Tier 3 |
| P5 | 1 | CMML-2 | *PTPN11* | NM_002834.3 | c.215C>T | p.Ala72Val | Missense | 43.9,  1094 | Tier 2 |
|  |  |  | *EZH2* | NM_004456.4 | c.2251C>T | p.Pro751Ser | Missense | 11.5,  348 | Tier 2 |
|  |  |  | *KMT2C* | NM_170606.2 | c.12140G>T | p.Ser4047Ile | Missense | 88.2,  569 | Tier 3 or  VUS |
|  |  |  | *KMT2C* | NM_170606.2 | c.2291C>T | p.Ser764Phe | Missense | 9.1,  1039 | Tier 3 |
|  | 2 | AML | *PTPN11* | NM_002834.3 | c.215C>T | p.Ala72Val | Missense | 2.4,  1094 | Tier 2 |
|  |  |  | *PTPN11* | NM_002834.3 | c.853T>C | p.Phe285Leu | Missense | 36.6,  1653 | Tier 2 |
|  |  |  | *PTPN11* | NM_002834.3 | c.182A>T | p.Asp61Val | Missense | 35.7,  2084 | Tier 2 |
|  |  |  | *EZH2* | NM_004456.4 | c.2251C>T | p.Pro751Ser | Missense | 16.5,  1289 | Tier 2 |
|  |  |  | *KMT2C* | NM_170606.2 | c.2291C>T | p.Ser764Phe | Missense | 8.1,  2010 | Tier 3 |
|  |  |  | *KMT2C* | NM_170606.2 | c.12140G>T | p.Ser4047Ile | Missense | 85,  1197 | Tier 3 or  VUS |
| P9* | 1 | MDS-MLD | *ECT2L* | NM_001077706.2 | c.2028+1_2028+14del | - | Splicing | 6.3,  605 | Tier 3 |
|  |  |  | *MPL* | NM_005373.2 | c.1775G>A | p.Arg592Gln | Missense | 15.8,  2712 | Tier 3 |
|  |  |  | *KMT2C* | NM_170606.2 | c.1181G>A | p.Cys394Tyr | Missense | 5.4,  5638 | Tier 3 |

Abbreviations: NGS, next-generation sequencing; NT, nucleotide; AA, amino acid; VAF, variant allele frequency; MDS-MLD, myelodysplastic syndrome with multilineage dysplasia; AML, acute myeloid leukemia; CMML-2, chronic myelomonocytic leukemia-2; VUS, variant of unknown significance

*Germline confirmation was performed using NGS data from skin fibroblast-derived DNA in some patients.

**Supplementary Table S3.** Patients diagnosed with AA who underwent follow-up bone marrow biopsy, chromosome karyotyping, and NGS.

| Control ID | Diagnosis | Age of Diagnosis | Follow-up Age | Chromosome test | NGS test |
| --- | --- | --- | --- | --- | --- |
| C1 | NSAA | 17Y | 33Y | 46,XX | WRAP53, c997_1003dup(p.Pro335LeufsTer24), 16.7% |
| C2 | NSAA | 5Y4M | 10Y4M | 46,XX | No tier 1/2 mutation |
| C3 | NSAA | 2Y6M | 4Y7M | 46,XY | No tier 1/2 mutation |
| C4 | SAA | 72Y | 74Y | 45,XY,-7 | No tier 1/2 mutation |
| C5 | NSAA | 36Y | 37Y | 46,XX | No tier 1/2 mutation |
| C6 | NSAA | 56Y | 64Y | 46,XY | TP53, c.404G>A(p.Cys135Tyr), 0.9% TET2, c.432del(p.Ser145ValfsTer7), 0.6% TET2, c.3442del(p.Tyr1148IlefsTer4), 0.2% |
| C7 | NSAA | 30Y | 60Y | 46,XX | No tier 1/2 mutation |
| C8 | NSAA | 49Y | 54Y | 46,XX | No tier 1/2 mutation |
